# Ataxia-telangiectasia in China: a case report of a novel *ATM* variant and literature review

**DOI:** 10.3389/fneur.2023.1228810

**Published:** 2023-07-26

**Authors:** Li Shao, Haoyi Wang, Jianbo Xu, Ming Qi, Zhaonan Yu, Jing Zhang

**Affiliations:** ^1^Department of Child Healthcare, Jinhua Maternity and Child Health Care Hospital, Jinhua, Zhejiang, China; ^2^Hangzhou D.A. Medical Laboratory, Hangzhou, Zhejiang, China; ^3^Central Laboratory, Precision Diagnosis and Treatment Center of Jinhua City, Jinhua, Zhejiang, China; ^4^Department of Laboratory Medicine, Jinhua Maternity and Child Health Care Hospital, Jinhua, Zhejiang, China; ^5^Department of Cell Biology and Medical Genetics, School of Medicine, Zhejiang University, Hangzhou, Zhejiang, China; ^6^Medical College of Tianjin University, Tianjin, China

**Keywords:** ataxia-telangiectasia, *ATM* gene, cerebellar atrophy, case report, literature review

## Abstract

**Background:**

Ataxia-telangiectasia (A-T) is a multisystem genetic disorder involving ataxia, oculocutaneous telangiectasia, and immunodeficiency caused by biallelic pathogenic variants in the *ATM* gene. To date, most *ATM* variants have been reported in the Caucasian population, and few studies have focused on the genotype–phenotype correlation of A-T in the Chinese population. We herein present a Chinese patient with A-T who carries compound heterozygous variants in the *ATM* gene and conducted a literature review for A-T in China.

**Case presentation:**

A 7-year-old Chinese girl presented with growth retardation, ataxia, medium ocular telangiectasia, cerebellar atrophy, and elevated serum alpha-fetoprotein (AFP) level, which supported the suspicion of A-T. Notably, the serum levels of immunoglobulins were all normal, ruling out immunodeficiency. Exome sequencing and Sanger sequencing revealed two likely pathogenic *ATM* variants, namely NM_000051.4: c.4195dup (p.Thr1399Asnfs^*^15) and c.6006 + 1G>T (p.?), which were inherited from her father and mother, respectively. From the Chinese literature review, we found that there was a marked delay in the diagnosis of A-T, and 38.9% (7/18) of A-T patients did not suffer from immunodeficiency in China. No genotype–phenotype correlation was observed in this group of A-T patients.

**Conclusion:**

These results extend the genotype spectrum of A-T in the Chinese population and imply that the diagnosis of A-T in China should be improved.

## Introduction

Ataxia-telangiectasia (A-T; OMIM no. 208900) is a rare multisystem disorder characterized by ataxia, oculocutaneous telangiectasia, global developmental delay, immunodeficiency, radiation hypersensitivity, and/or increased susceptibility to malignancy particularly of lymphoid origin (1–3). This condition is caused by biallelic pathogenic variants in the *ATM* gene located at chromosome 11q22.3 (1–3). The phenotype of A-T is associated with some degree of preservation of ATM kinase activity. The classical form of A-T is a severe multisystem disorder with an early onset, progressive, and neurodegenerative course due to biallelic loss-of-function variants in the *ATM* gene, whereas the milder type of A-T, characterized by slow neurological progression and/or later onset and designated “variant A-T”, is caused by biallelic variants with at least one non-truncating variant (missense or splice site variant) resulting in the presence of residual ATM kinase activity (3). The *ATM* gene harbors 66 exons and encodes a PI3K-family kinase with 3,050 amino acid residues. Hitherto, more than 1,400 unique variants have been identified in the *ATM* gene (https://www.lovd.nl/ATM).

However, studies investigating *ATM* variants and A-T in China are scarce (4–9). Herein, we describe a Chinese female patient affected by A-T with compound heterozygous variants in the *ATM* gene, one of which is novel. Furthermore, we review the literature on the genotype–phenotype spectrum of A-T in the Chinese population.

## Case presentation

### Clinical report

**Figure 1:**
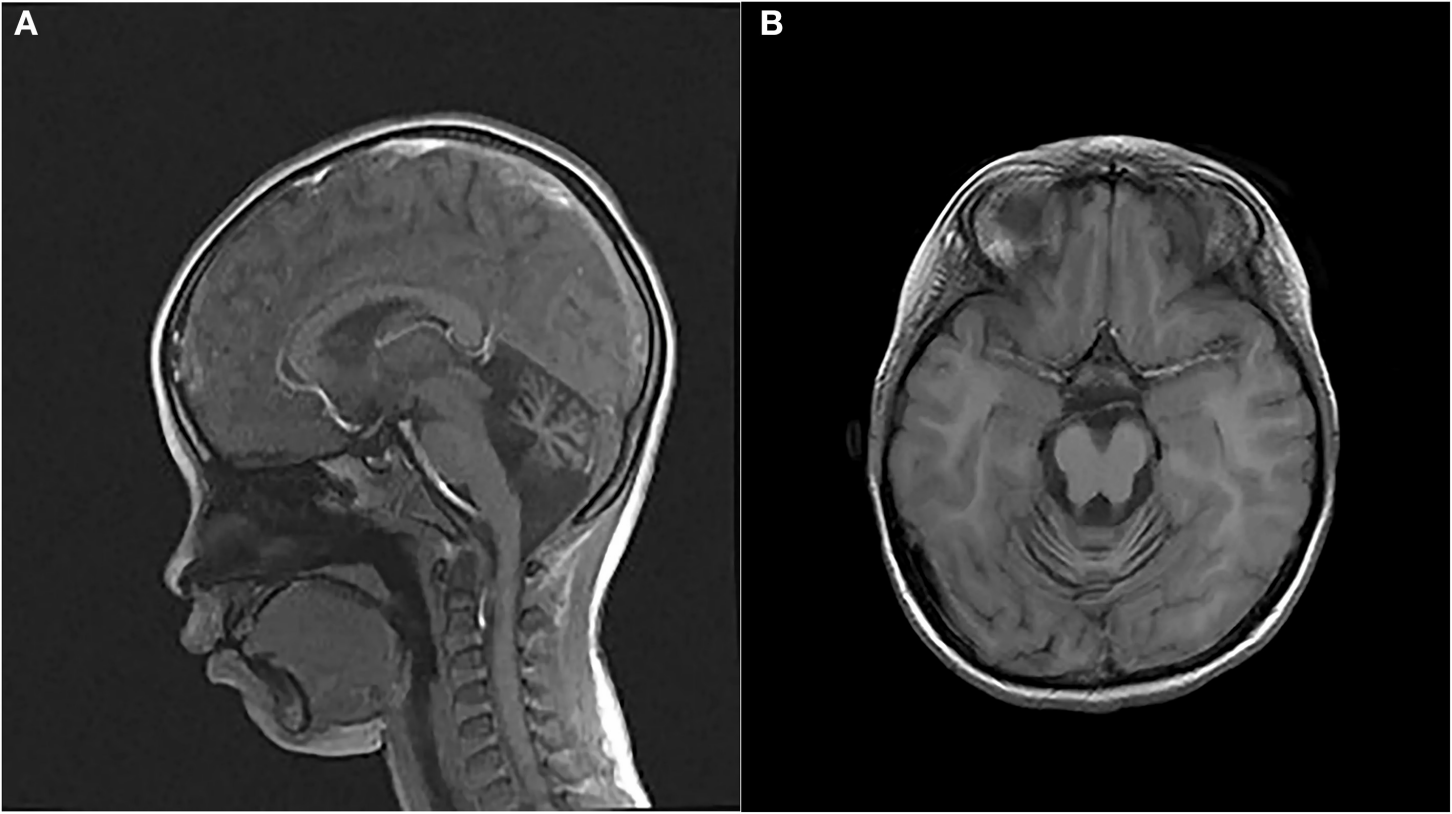
Magnetic resonance images (MRIs) of the patient's brain. **(A)** Sagittal T2-weighted brain MRI. **(B)** Axial T1-weighted brain MRI.

The proband, a 7-year-old Chinese girl, was referred to our hospital for the evaluation of growth retardation. Her weight and height were below the 3rd percentile, and she exhibited medium ocular telangiectasia since the age of 5. Neurological examinations revealed progressive limb and truncal ataxia, diagnosed on the basis of gait instability, dysarthria, spasticity, and delayed cognitive and motor development. At the last follow-up visit, the 9-year-old patient was not wheelchair-bound. The parents reported that the proband initially developed symptoms of ataxia at the age of 2. Laboratory tests showed that the serum levels of α-fetoprotein (AFP), lactic dehydrogenase (LDH), and carbohydrate antigen 199 (CA199) were significantly elevated, whereas the serum levels of carcinoembryonic antigen (CEA) and immunoglobulins (IgA, IgG, IgM, and IgE) levels were within the normal range (Table 1). Brain magnetic resonance imaging (MRI) unveiled cerebellar atrophy with the enlargement of cerebellar sulci (Figure 1). The parents were both normal, and no relatives were reported to have neurodegenerative diseases except for the grandpa's cousin who presented amentia. A-T was suspected based on the clinical features of ataxia, cerebellar atrophy, ocular telangiectasia, and elevated serum AFP level. Given the absence of immunodeficiency and the conservative wishes of the parents, we decided to follow up with the patient at present. Medical interventions will not be administered until the disease progresses.

**Table 1:** Clinical and laboratory features of the patient.

	**Ataxia-age at onset (years)**	**Telangiectasias-age at onset (years)**	**Age at diagnosis (years)**	**Cerebellar atrophy**	**Serum AFP (ng/ml)**	**Serum CA199 (U/ml)**	**Serum LDH (U/L)**	**Serum CEA (ng/ml)**	**IgA (g/L)**	**IgG (g/L)**	**IgM (g/L)**	**IgE (kU/L)**
Patient	2	5	7	Atrophied	319.61	52.73	283	<0.5	1.48	12.8	1.8	<2.0
Normal value					0–8.1	0–37	109–245	0–5	0.70–4.00	7.00–16.00	0.40–2.30	<100

AFP, α-fetoprotein; LDH, lactic dehydrogenase; CA199, carbohydrate antigen 199; CEA, carcinoembryonic antigen.

### Genetic analysis

**Figure 2:**
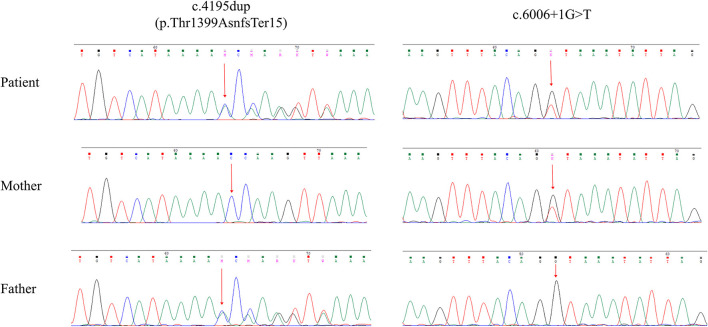
Sanger sequencing validation of the *ATM* variants of all family members.

To validate the clinical suspicion of A-T, exome sequencing was performed using the peripheral blood from the patient. The procedures of genetic analysis and the filtering condition for variants were described by Shalash et al. (10). We identified compound heterozygous variants in the *ATM* gene, NM_000051.4 (*ATM*): c.4195dup and c.6006 + 1G>T. The intronic variant c.6006 + 1G>T (ClinVar Variation ID: 2029577) was expected to disrupt RNA splicing by affecting a donor splice site in intron 40 of the *ATM* gene, thereby it was classified as pathogenic in the ClinVar database. The variant c.4195dup located at exon 28 led to a frameshift at residue 1399, which was predicted to produce a truncated protein of 1,412 amino acids (p.Thr1399Asnfs^*^15) lacking the FAT, PI3K/PI4K catalytic, and FATC domain. The CADD and GERP scores were 33 and 4.22, respectively, both of which exceeded the threshold for pathogenicity. Using the MutationTaster tool, both variants were predicted to be disease causing. Familial segregation analysis was verified using Sanger sequencing. The compound heterozygous variants, c.4195dup and c.6006 + 1G>T, were inherited from the unaffected father and mother, respectively (Figure 2). The frameshift variant c.4195dup was not included in the gnomeAD, HGMD, and 1000G databases, thereby confirming its novelty. We did not identify any pathogenic variants in other nervous system-related genes.

### Chinese literature review

Seventeen Chinese cases with A-T from six studies were collected to reveal the genotypic and phenotypic features of A-T in China (Table 2) (4–9). Combined with the case in this article, we found that there was a delay of ≥5 years in diagnosis since the onset of clinical presentations in most cases. With regard to the *ATM* genotype, compound heterozygosity is the major allelic type in China possibly due to the legal prohibition of consanguineous marriage in our country. Most cases had at least one *ATM* truncated (frameshift or non-sense) variant, which is in agreement with the classical clinical signs of A-T. Seven cases did not present immunodeficiency, which is one of the cardinal clinical indications in A-T. No genotype–phenotype correlation was found in this group of A-T patients.

**Table 2:** Literature review for Chinese patients with ataxia-telangiectasia.

**Family**	**Patient**	**Nucleotide change**	**Protein effect**	**Allele**	**Ataxia**	**Telangiectasias**	**Cerebellar atrophy**	**Elevated α-fetoprotein**	**Immune manifestations**	**Ataxia-age at onset (years)**	**Telangiectasias-age at onset (years)**	**Age at diagnosis (years)**	**References**
I	1	c.1346G>C	p.Gly449Ala	hom	+ (severe)	+	+	+	IgA deficiency and T-cell defect	7	7	15	(4)
II	2	c.610G>T and c.6679C>T	p.Gly204^*^ and p.Arg2227Cys	CH	+ (moderate)	+	+	+	agammaglobulinemia and T-cell defect	10	10	15	
III	3	c.1464G>A and c.56–1G>A	p.Trp448^*^ and Splice acceptor	CH	+	+	+	+	IgG deficiency	2	3	13	(5)
	4									1.5	2	4	
IV	5	c.2680delG and c.7166C>G	p.Asp894 Ilefs^*^4 and p.Ser2389^*^	CH	+	+	+	+	-	2.5	3	14	
	6									1.5	1	7	
V	7	c.3174G>A and Exon 63 deletion	p.Trp1058^*^ and N/A	CH	+	+	+	+	-	4	1	13	
VI	8	c.2152_2154 delinsAAAC and c.8713_ 8714insCA	p.Cys718Lysfs^*^19 and p.Val2906Glnfs^*^32	CH	+	+	+	+	-	1.5	2	8	
VII	9	c1402_1403delAA and c.2413C>T	p.Lys468Glufs^*^17 and p.Arg805^*^	CH	+	+	+	+	IgG deficiency	2	2	8	
VIII	10	c.6885G>T and c.3742_3743 insGGAGGTTCT	p.Val1248Phe and p.Tyr1248Trpfs^*^10	CH	+	+	+	+	-	2	2	7	
IX	11	c.50_72 + 7del	p.Asp18_L ys24delins(23)	hom	+(severe)	+	+	+	-	2.5	NR	13	(6)
	12				+ (moderate)	+	+	+	High IgM	6.5	NR	12	
	13				+ (moderate)	+	+	+	-	6.5	NR	11	
X	14	c.5939–5948del and c.2639–384A>G	p. Ile980fs and p.Gly880 Glufs^*^14	CH	+ (moderate)	+	-	NR	Low IgA and IgG, high IgM, B- and T-cell lymphopenia	7	7	7	(7)
	15				+ (severe)	+	+	NR	Mild lymphopenia with IgA defect	5	6	15	
XI	16	c.6679C>T and c.5773delG	p.Arg2227Cys and p.Gly1925 Glufs^*^12	CH	+	+	+	+	Increased leukocyte count and agammaglobulinemia	2	NR	7	(8)
XII	17	c.1402_1403delAA	p.Lys468Glufs^*^18	hom	+ (severe)	+	+	+	Low IgA and IgG	2	4	10	(9)
XIII	18	c.4195dup and c.6006 + 1G>T	p.Thr1399Asnfs^*^15 and splice donor	CH	+	+	+	+	-	2	5	7	This study

+, present; -, not present; NR, not reported; ho, homozygosity; CH, compound heterozygosity. ^*^ indicates termination codon.

## Discussion

We report a Chinese patient affected by A-T with compound heterozygous *ATM* genotype; one of the identified variants is unreported. In China, patients are suspected of A-T only when classical clinical signs, namely ataxia and oculocutaneous telangiectasia are present. However, the onset age of ataxia and oculocutaneous telangiectasia is sometimes different. Some manifestations may even occur prior to ataxia and oculocutaneous telangiectasia, such as recurrent infections of unknown origins (7). These factors, combined with the extremely low prevalence of A-T at 1:40,000–1:300,000 (3), collectively lead to a delay in the diagnosis of A-T in China. According to the Chinese literature review (Table 2), it takes more than 5 years in China to complete the diagnosis of A-T since the onset of symptoms; therefore, great efforts are still warranted to ameliorate the diagnosis of this rare genetic disorder. Devaney et al. (11) reported the presentation and diagnostic delay in A-T, which further consolidated our conclusions.

The early measurement of serum AFP is an easily detectable and reliable biological hallmark of A-T (3). As expected, the serum AFP level is elevated in all reported Chinese patients with A-T (Table 2), except for two patients whose AFP levels were not reported (7). Immunodeficiency is also an important indicator for A-T, which is associated with ATM protein dysfunction in immunoglobulin class-switch recombination (CSR), V(D)J recombination, and B- and T-cell homeostasis (12, 13). It has been reported that immunodeficiency is present in approximately two-thirds of A-T patients (1, 3). The Chinese literature review (Table 2) shows that 38.9% (7/18) of Chinese patients with A-T, including the one in our case, have normal immune status at the time of diagnosis, which is in agreement with the literature data. For these patients who have no signs of immunodeficiency, elevated serum AFP levels and neurological manifestations are the most suggestive.

## Conclusion

In conclusion, our findings extend the genotype spectrum of A-T in the Chinese population and suggest that the diagnosis of A-T in China should be improved in clinical practice. The limitations of our study are the small number of patients and a lack of functional investigation.

## Data availability statement

The datasets presented in this article are not readily available because of ethical and privacy restrictions. Requests to access the datasets should be directed to the corresponding authors.

## Ethics statement

The studies involving human participants were reviewed and approved by the Ethics Committee of Jinhua Maternity and Child Health Care Hospital. Written informed consent to participate in this study was provided by the participants' legal guardian/next of kin. Written informed consent was obtained from the individual(s) and minor(s)' legal guardian/next of kin, for the publication of any potentially identifiable images or data included in this article. Written informed consent was obtained from the participant/patient(s) for the publication of this case report.

## Author contributions

JX, LS, and JZ were the patient's physicians. HW and MQ reviewed the literature and contributed to manuscript drafting. HW and ZY performed the mutation analysis. JX and JZ conceptualized and designed the study, coordinated and supervised data collection, and critically reviewed the manuscript for important intellectual content. HW, ZY, and JZ were responsible for the revision of the manuscript for important intellectual content. All authors contributed to the article and approved the submitted version.
